# Safety and Effectiveness Outcomes of a Novel Automated Titanium Suture Fastener Device Applied for Heart Valve Surgery in an Ovine Model

**DOI:** 10.3389/fcvm.2022.783208

**Published:** 2022-02-11

**Authors:** Bin Li, Shanshan Bai, Guangxin Yue, Jinyan Zhu, Min Zhang, Baiqing Yang, Jiafei Luo, Yang Sun, Leisheng Zhang, Xin Wang

**Affiliations:** ^1^Beijing Key Laboratory of Preclinical Research and Evaluation for Cardiovascular Implant Materials, State Key Laboratory of Cardiovascular Disease, Animal Experimental Centre, National Centre for Cardiovascular Disease, Fuwai Hospital, Chinese Academy of Medical Sciences and Peking Union Medical College, Beijing, China; ^2^Department of Blood Products and Substitutes, Beijing Institute of Transfusion Medicine, Beijing, China; ^3^Department of Pathology, National Centre for Cardiovascular Disease, Fuwai Hospital, Chinese Academy of Medical Sciences and Peking Union Medical College, Beijing, China; ^4^Shandong Provincial Key Laboratory of Translational Medicine for Rheumatic and Immune Diseases, Qianfoshan Hospital, The First Affiliated Hospital of Shandong First Medical University, Jinan, China; ^5^The Postdoctoral Research Station, School of Medicine, Nankai University, Tianjin, China

**Keywords:** preclinical evaluation, Easy-Knot, safety and effectiveness, annuloplasty ring implantation, cardiac surgery

## Abstract

**Objective:**

This study was designed to evaluate the operability, effectiveness, and safety of the automated titanium suture fastener in a preclinical ovine model in comparison with manual tying in a mitral valve annuloplasty ring implantation surgery.

**Methods:**

Eighteen adult Small-tailed Han sheep were prepared for the surgery of mitral valve annuloplasty ring implantation through lateral thoracotomy under cardiopulmonary bypass (CBP). A total of 12 stitches were performed to secure an annuloplasty ring, with 6 stitches done with the automated fastener and the other 6 by manual tying. The knotting time for the automated fastener or manual tying was recorded, respectively. The firmness of knots, mitral valve integrity, biocompatibility, thrombosis, local reactions, and other aspects were also compared at follow-up time (Days 30, 60, 90, and 180).

**Results:**

Of the 18 sheep, 16 survived to the designated endpoints and were enrolled for further analysis. Compared with the control group, the knotting time was significantly reduced with the automated fastener (*p* < 0.01). All the annuloplasty rings were tightly secured by 6 fastener clips and 6 hand-made knots without any disengagement or displacement. All the mitral valves were intact without any defect, stenosis, prolapse, valve insufficiency, or perforation. Endothelialization was comparable between the two groups by Day 60. Small red thrombi formed at the thread end of the suture in both groups. No thrombus was found on the surface of the titanium clip. All the thrombi were within the acceptable range for the antithrombotic property. Thrombosis showed no significant difference by Day 60. No significant differences in the inflammatory response and pathological lesions were observed by Day 60. One case of diffuse renal infarction (area ratio = 20%) and 1 case of small focal renal infarction (area ratio < 5%) were caused by thromboembolism.

**Conclusions:**

The automated fastener significantly shortened the procedure time of tying knots for the implantation of the annuloplasty ring in the ovine model, with comparable safety and effectiveness as manual tying.

## Introduction

Surgical procedures for mitral valve repair and replacement under cardiopulmonary bypass (CPB) remain a superior treatment option for patients with severe valvular heart disease (VHD) (1). It is well-established that the surgical procedure could be extremely complicated and with potential ensued complications of CPB. However, CPB remains unavoidable for the vast majority of cardiac surgery and prolonged CPB time and aortic cross-clamp (ACC) time are reported to be independently associated with postoperative morbidity and mortality (2–4).

New techniques and technologies have been introduced to reduce CPB and ACC times. Among the new adjunct devices, an automated titanium suture fastener is the most widely accepted by cardiac surgeons (5). This device is designed to enable quick, reliable, and easy suture fastening with a single squeeze. The advantage of an automated fastener has been demonstrated by an *ex vivo* study (6) and multiple clinical studies (7–10) that the adoption of fastener significantly reduced CPB and ACC times. However, the incidence of valvular regurgitation (11, 12)and leaflet perforation (13, 14) by using a fastener has been reported. Yet, the safety and effectiveness of the fastener have not been fully investigated in preclinical *in vivo* studies, whether any of the existing complications are related to the fastener could not be determined. In addition, the use of fastener translating to the improved patient outcome remains debatable (15, 16).

Herein, we designed an ovine self-control experiment, that an annuloplasty ring was secured by an automated fastener on the left half side and manual tying on the right, to evaluate the safety and effectiveness outcomes of an automated fastener (Easy-Knot®, Maidifeng Medical Technology Co. Ltd, Beijing, China). In this model, we mainly focused on the evaluation of firmness of knots, mitral valve integrity, biocompatibility, thrombosis, local reactions, and other aspects, providing a reference evaluation system for an automated titanium suture fastener or similar devices in cardiac valvular surgery.

## METHODS

### Animals and Ethical Approval

A total of 18 Small-tailed Han sheep (16 female sheep and 2 male sheep) weighing between 40 and 60 kg were procured from Laboratory Animal Center, Fuwai Hospital, Chinese Academy of Medical Sciences & Peking Union Medical College. The basic information of the animals is listed in Supplementary Table S1. This study adopted the self-controlled method, the fixation of mitral annuloplasty ring by either the novel titanium fastener or the traditional manual knotting was conducted in the same individual. All procedures of the experiment were approved by the Ethical Committee of Fuwai Hospital, Chinese Academy of Medical Sciences & Peking Union Medical College (Approval No. 0097-2-18-HX (X) and in accordance with the Standard Operation Procedures (SOP) as we previously described (17, 18).

### The Application of a Novel Sterile Titanium Clip Device

**Figure 1:**
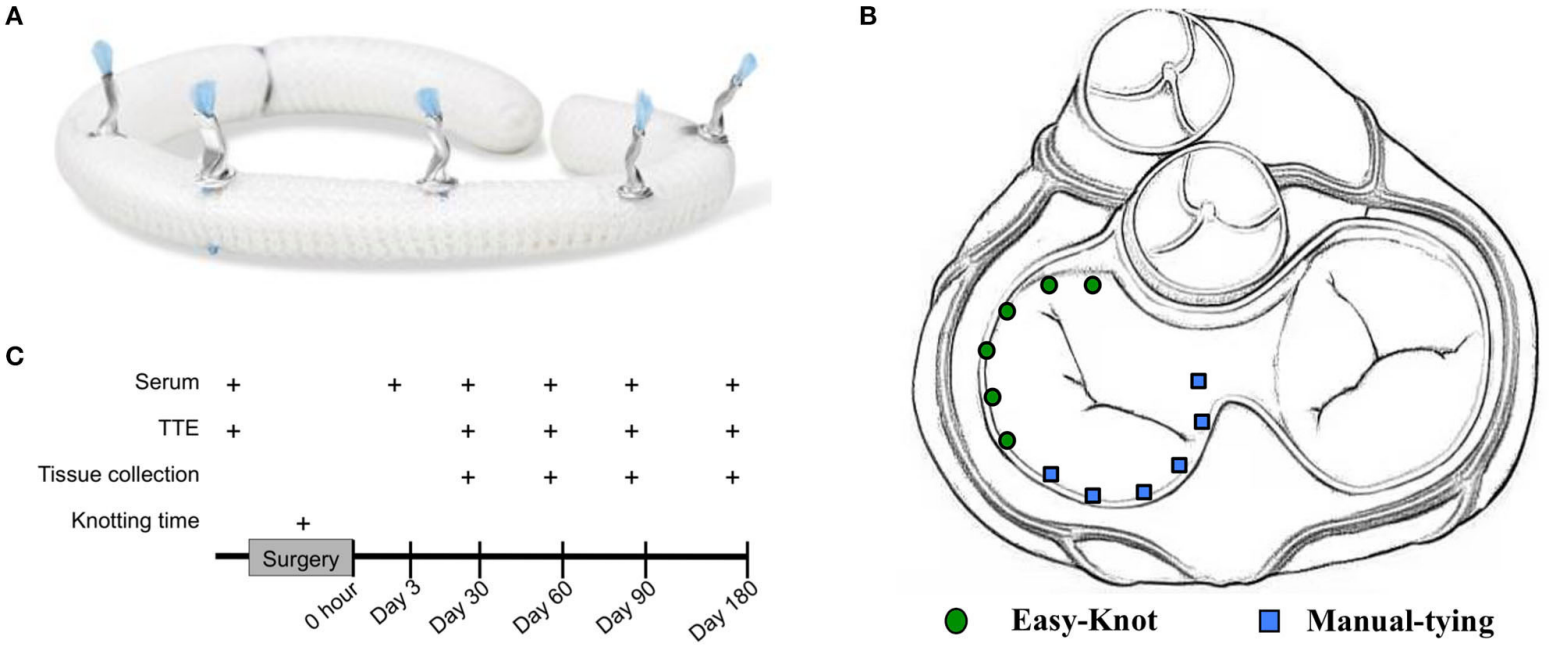
Experimental design and knotting effect of Easy-Knot. **(A)** Realistic graph of knots on an annuloplasty ring tied by Easy-Knot. **(B)** Schematic diagram of knotting sites by Easy-Knot (green dot) and manual tying (blue square) for annuloplasty ring fixation. **(C)** Data acquisition guideline.

The Easy-Knot® device, which is a class III medical appliance, consists of a sterile titanium clip and a disposable sterile fastener, provided by Beijing Maidifeng Medical Technology Co. Ltd, Beijing, China. The titanium clip is the implanted part for the fixation of each stitch. The working principle is that after sewing, the titanium clip is put surrounding the suture and then squeezed and bent over by the fastener to secure and fix the suture. The blade is placed in the front and then cut off the excessive suture. The knotting effect is shown in Figure 1A.

### Animal Anesthesia

Before the surgical operation, the sheep fasted for 24 h and water deprived for 8 h. The sheep were then incipiently narcotized with 4 mg/kg disoprofol (WZS-50F6), followed by weighing, skin preparation, and body surface ultrasound. The sheep were sent to the operation room for surgery after endotracheal intubation. During the operation, Isoflurane was used for the maintenance of anesthesia with the anesthetic equipment (Drager Primus, Germany).

### Surgical Procedure

The sheep were put in the right decubitus position on the operating table and disinfected with iodophor disinfectant. The lateral thoracotomy was carried out between the 3rd and 4th ribs. The pericardium was cut-through (COVIDIEN Force FX-8C, USA), and followed by traction and suspension to establish CPB (STOCKERT SC, Germany). The left atrium was opened and the mitral valve was exposed after cardiac arrest. As shown in Figure 1B, 12 stitches were performed to secure the annuloplasty ring, with 6 stitches done by using the fastener (experimental group) and the other 6 by traditional manual knotting (control group). For each group, 2 stitches were on the anterior leaflet, and 4 on the posterior leaflet. The knotting time was recorded for each group. During the operation, the ECG, arterial blood pressure (Philips, MP60, The Netherlands), heart rate (Philips IntelliVue MP70), body temperature, blood gas (Abbott i-STAT1, IL, USA), and activated clotting time (ACT) (HEMOCHRON Jr Signature) were closely monitored. The drainage tube was placed between the 6th and 7th intercostal muscles for liquid drainage after surgery.

### Postoperative Care

Postoperative care, such as postoperative intensive care unit (ICU) nursing, ventilator assisted breathing, continuous monitoring of blood pressure, and ECG (Philips, MP60) were performed to maintain circulation stability, and vasoactive drugs were given when necessary following the postoperative standard operating procedures. Once the sheep gradually recovered and resumed autonomous respiration, the ventilator and endotracheal intubation were removed and replaced by nasal oxygen assistance. The pleural drainage tube was removed when the liquid drainage volume dropped under 20 ml/h.

After regaining consciousness, the sheep were transferred to the feeding area, and diet and water consumption were strictly controlled for 1 week after the operation. The mental state, appetite, respiration, wound complications, and other potentially adverse symptoms of the animals were carefully observed daily, and the surgical incisions were disinfected.

### Postoperative Anti-infection

Postoperative anti-infection was conducted to prevent sheep from secondary infection and the resultant death. Specifically, 4.8 million unit's penicillin and 0.2 g amikacin per time was intramuscularly injected two times per day for continuous 7 days. Anticoagulant treatment consisted of Aspirin (100 mg per time, orally, once a day) and Warfarin (6 mg per time, orally, once a day) was given from the day after surgery to the end of the experiment. Low molecular weight heparin (6,000 IU per time, subcutaneously, two times a day) was injected immediately after surgery and the following 3 days.

### Sampling

The sampling guideline was shown in Figure 1C. Briefly, the knotting time was recorded during the surgery. Routine tests of the surgery, such as blood tests and transthoracic echocardiography (TTE) (GE E9, IL, USA) were performed at indicated time points. Serum was isolated from the whole blood and tested for blood routine, myocardial zymogram, liver function, kidney function, and coagulation, individually. Animals were randomly assigned for Day 30, 60, 90, and 180 groups. The animals were sacrificed at the designated endpoints and tissues, such as heart, liver, spleen, lung, kidney, and brain were collected for further analysis.

### Scanning Electron Microscope (SEM)

Scanning electron microscope (SEM) analysis was performed to evaluate the endothelialization as we recently reported (18). Briefly, the cardiac tissues on the knotting site were fixed with 2.5% glutaraldehyde (v/v) and 1% osmic acid (v/v) (Sigma-Aldrich, St Louis, MO, USA), and followed by dehydration with gradient alcohol and isoamyl acetate. After that, the samples were dried in a critical point dryer and sprayed by a vacuum ion. The ultrastructure of the samples was observed by using the SEM (JSM-5050, Japan), such as the fibrin and platelet deposition, the extent of new intima growth, and thrombosis. The endothelialization was calculated based on 5 random fields of view according to the formula: endothelialization % = (neointimal area/material area) × 100%.

### Hematoxylin-Eosin (H&E) and Masson Staining

Histology was conducted as we recently described (19). Briefly, samples were collected from each organ, such as the left and right ventricles of the heart and ventricular septum, bilateral lungs, liver, spleen, kidneys, and cerebellum. After paraffin embedding and sectioning, H&E staining and Masson trichrome staining were performed to assess pathological findings and fibrosis, respectively. Images were captured by Olympus BX51 microscope (Olympus Corporation, Japan) and analyzed with Image-Pro Plus6.0 Image software. Fibrotic thickness was further measured with the ZEN Image software.

### Safety Evaluation

The safety of the novel sterile titanium clip device was evaluated from the following five aspects: (1) firmness of the knot and mitral valve integrity were tested by the occurrence of loosening or missing titanium clips, peri-valvular leakage, defects, stenosis, prolapse, incomplete closure, perforation, and excrescence at the end points through gross examination. (2) Biocompatibility was evaluated by endothelialization through SEM. (3) Thrombosis rate was evaluated according to the formula: (number of knots with thrombus/number of total knots) x 100%. The thrombus size was graded according to the biological test methods “GB/T 14233.2-2005” Appendix B (Supplementary Table S2). (4) Local reactions were evaluated by the occurrence of inflammatory response and pathological lesions according to the professional standards (GB/T16886.6- 2015/ISO10993-6: 2007). Briefly, inflammatory cells were counted and graded based on 3 random fields of view of H&E staining slides. The total score of 7 items, such as (i) neutrophils, (ii) lymphocytes, (iii) eosinophils, (iv) plasma cells, (v) giant cells, (vi) macrophages, and (vii) tissue necrosis (0–4 points for each item) was calculated to evaluate inflammation reaction. The total score of 4 items, such as (i) neovascularization, (ii) fibrosis, (iii) fat infiltration, and (iv) calcification (0–4 points for each item) was calculated to evaluate pathological lesions. (5) Fibrotic thickness was calculated based on Masson trichrome staining.

### Statistical Analyses

All data in the study were statistically analyzed by utilizing the SPSS 16.0 software. Repeated measures ANOVA was used between continuous variables conforming to normality and shown as mean ± SD. Mann–Whitney *U* was used between data groups not conforming to normality and ordinal variables and shown as median ± interquartile (IQ). The value of *p* < 0.05 was considered statistically significant. ^*^, *p* < 0.05; ^**^, *p* < 0.01; ns, not significant.

## Results

### Implantation Surgery and Survival

Of the 18 sheep, two unexpected deaths occurred. One sheep died during the operation due to atrial septal deficiency (ASD). An air intake occurred at the atrial septal defect and CBP was stopped during atrial septal repair, resulting in arrhythmia and heart failure. The pathological manifestation showed bleeding spots on the surface of the heart, multiple bleeding in the myocardium, congestion and bleeding in both lungs. The other sheep died 171 days after the operation. The cause of death was the prolonged operation time intending to fix the mismatch between the annuloplasty ring and the animal annulus. Furthermore, postoperative moderate mitral regurgitation, slow heart rate, eating less, diarrhea, excessive consumption of physical function, and blood stasis in both lungs were also observed. Together, the causes of deaths were unrelated to the experimental device. A total of 16 sheep (Supplementary Table S1) reached the designated endpoints without complications and were enrolled for further analyses. Animals were sacrificed after 30 days (*n* = 3), 60 days (*n* = 3), 90 days (*n* = 4), and 180 days (*n* = 6) follow-up time.

**Figure 2:**
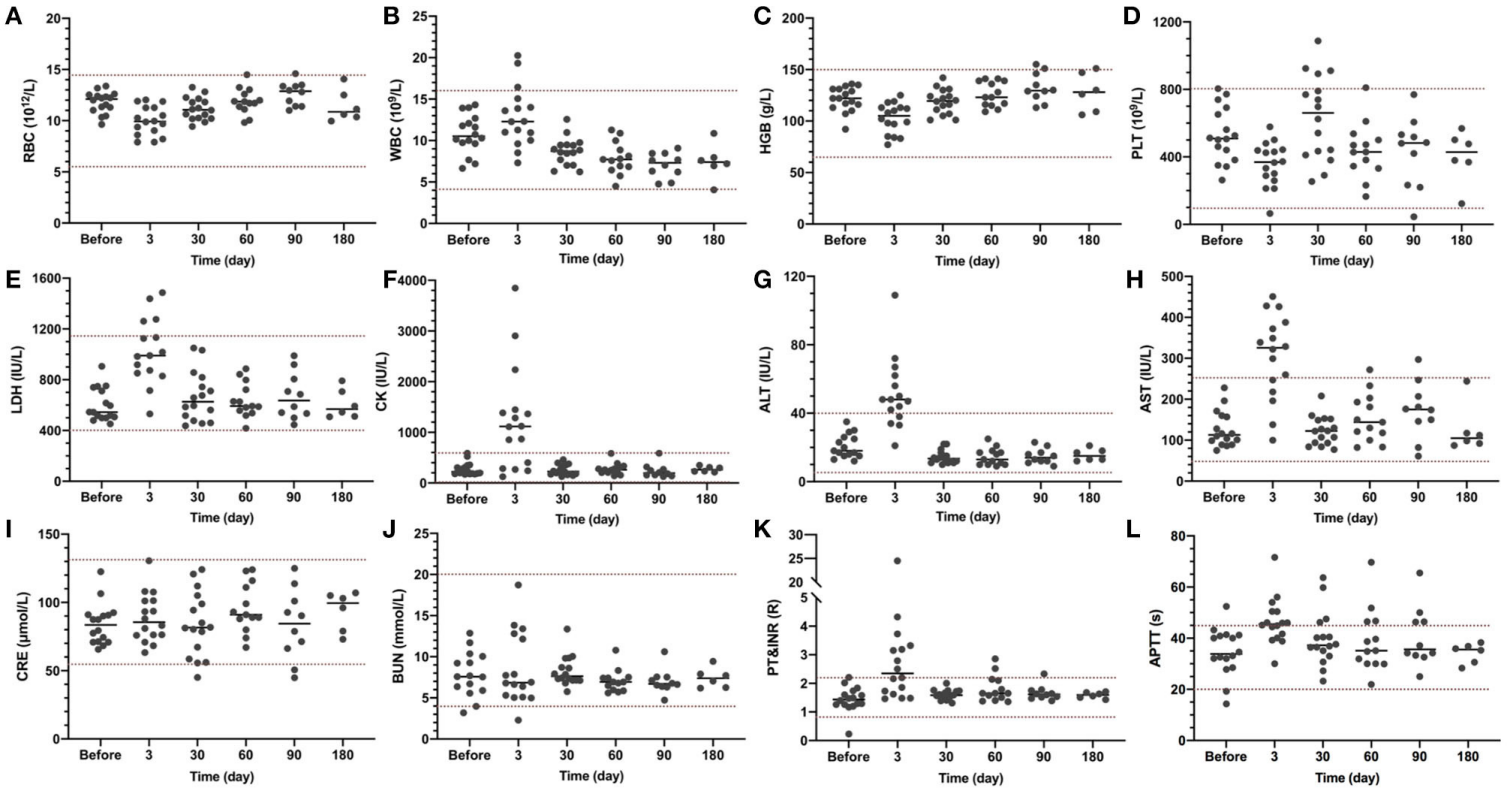
Blood test results at indicated time points. Blood routine, myocardial zymogram, liver function, kidney function, and coagulation were examined. The normal range is indicated between the two dash lines. **(A)** RBC, red blood cell count; **(B)**. WBC, white blood cell count; **(C)** HGB, hemoglobin; **(D)** PLT, platelet; **(E)** LDH, lactic dehydrogenase; **(F)** CK, creatine kinase; **(G)** ALT, alanine aminotransferase; **(H)** AST, aspartate transaminase; **(I)** CRE, creatinine; **(J)** BUN, blood urea nitrogen; **(K)** PT, prothrombin time; INR, international normalized ratio; and **(L)** APTT, activated partial thromboplastin time.

Blood tests and transthoracic echocardiography (TTE) were introduced as routine tests of the surgery. As shown in Figure 2, short-term postoperative hemoglobin (Figure 2C) and platelet counts (Figure 2D) were within the normal range, indicating no significant hemolysis occurred during the operation. Serum myocardial zymogram (Figures 2E,F) and liver function (Figures 2G,H) exceeded the normal ranges at Day 3 and returned back to normal at Day 30, which was normal for cardiac surgeries. Renal function was not affected during the entire experiment (Figures 2I,J). The slight coagulation disorder was caused by the postoperative anti-infection treatment: the longer prothrombin time (Figure 2K) at Day 30 was due to the heparin and the postoperative activated partial thromboplastin time (Figure 2L) exceeding the normal range was due to the Warfarin.

**Figure 3:**
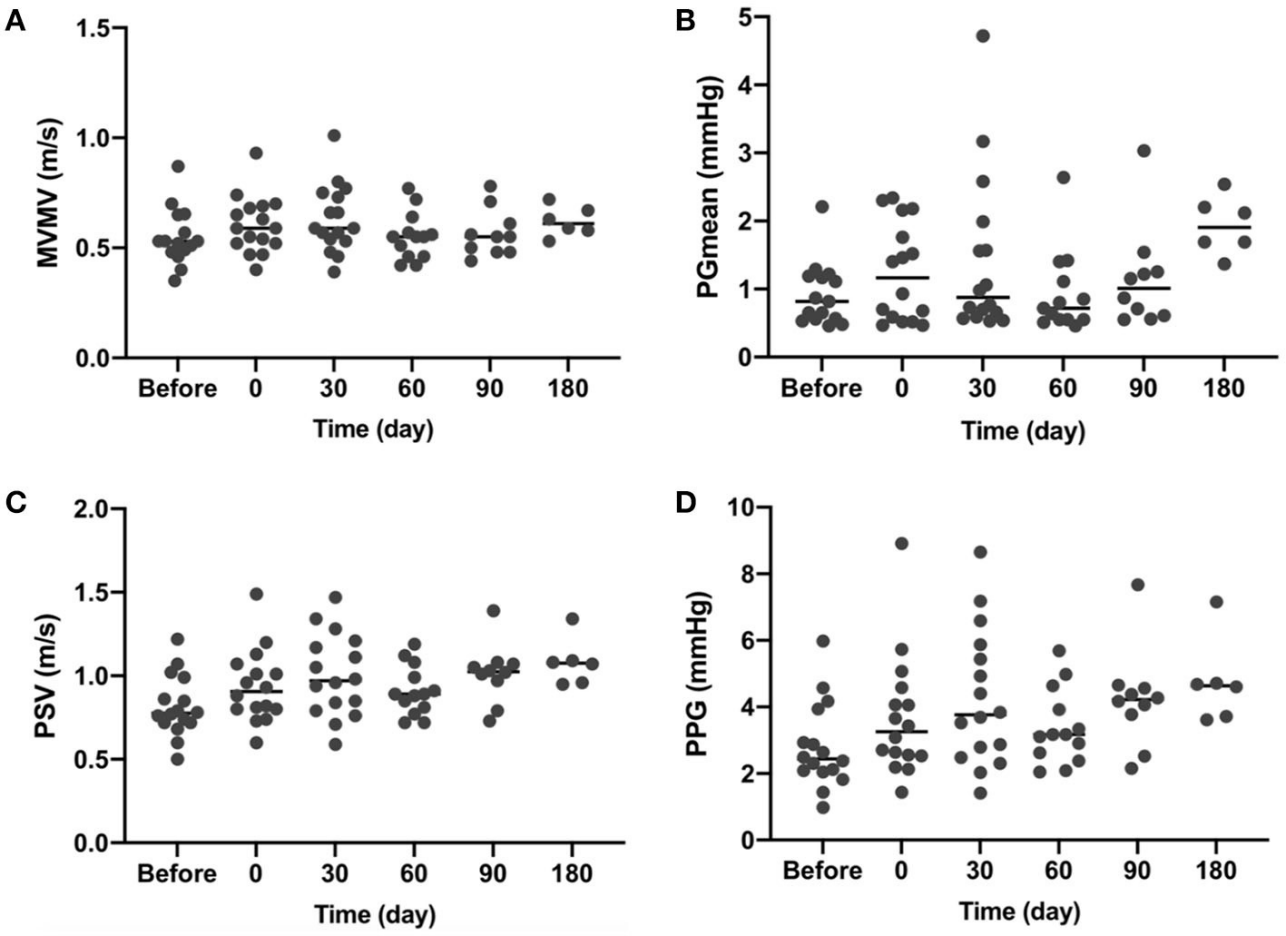
Transthoracic echocardiography (TTE) results at indicated time points. **(A)** MVMV, mean velocity of the mitral valve; **(B)** PGmean, mean pressure gradient; **(C)** PSV, peak systolic velocity; and **(D)** PPG, a peak pressure gradient.

As the annuloplasty ring was implanted in healthy sheep without any preoperative mitral insufficiency, the mitral valve would close even more tightly after implantation. As shown in Figure 3, mean pressure gradient (Figure 3B), postoperative peak systolic velocity (Figure 3C), and peak pressure gradient (Figure 3D) were increased due to the more tightly closed mitral valve. Slight to moderate mitral regurgitation was observed in 4 sheep short-term after surgery. No mitral regurgitation was observed at a longer follow-up time. All the valves functioned well without pathological changes at the endpoints.

### The Operability Outcomes of the Easy-Knot

For each enrolled sheep, twelve stitches were performed to fix an annuloplasty ring, with 6 stitches done by using the sterile automated titanium fastener device, namely the Easy-Knot (experimental group), and the other 6 by traditional manual tying (control group). For each group, 2 stitches were on the anterior leaflet, and 4 on the posterior leaflet (Figure 1C). A total of 96 knots were tied by the Easy-Knot and no loosen or fallen off knots were found by the endpoints. In general, the Easy-Knot is an effective and easy-to-use device.

### The Effectiveness Outcomes of the Easy-Knot

To assess whether the Easy-Knot could tie secure knots in a shorter time, the knotting time of 6 knots tied by either the Easy-Knot or manually was recorded individually. As shown in Table 1, tying 6 knots with the Easy-Knot took 157.31 ± 58.84 s, while manual tying cost 345.56 ± 68.68 s, which means making one knot with the Easy-Knot took only around 30 s (26.22 ± 9.81 s) on average, which is significantly shorter than manual tying (57.59 ± 11.45 s).

**Table 1:** Calculation of the total and average knotting and cutting time during heart valve surgery.

**Items**	**The control group** **(***n =*** 96)**	**The experimental group** **(***n =*** 96)**	**P-value**
Total knotting and cutting time (s)	345.56 ± 68.68	157.31 ± 58.84	<0.001^***^
Average knotting and cutting time (s)	57.59 ± 11.45	26.22 ± 9.81	<0.001^***^

*Mean ± SD*,

*** *P < 0.001*.

The successful implantation and proper knotting sites were confirmed by gross examination at the endpoints. All the annuloplasty rings were secured tightly without any disengagement or displacement. Thus, the Easy-Knot significantly shortened the knotting time without compromising the firmness of the knots and operational accuracy.

### The Safety Outcomes of the Easy-Knot

**Figure 4:**
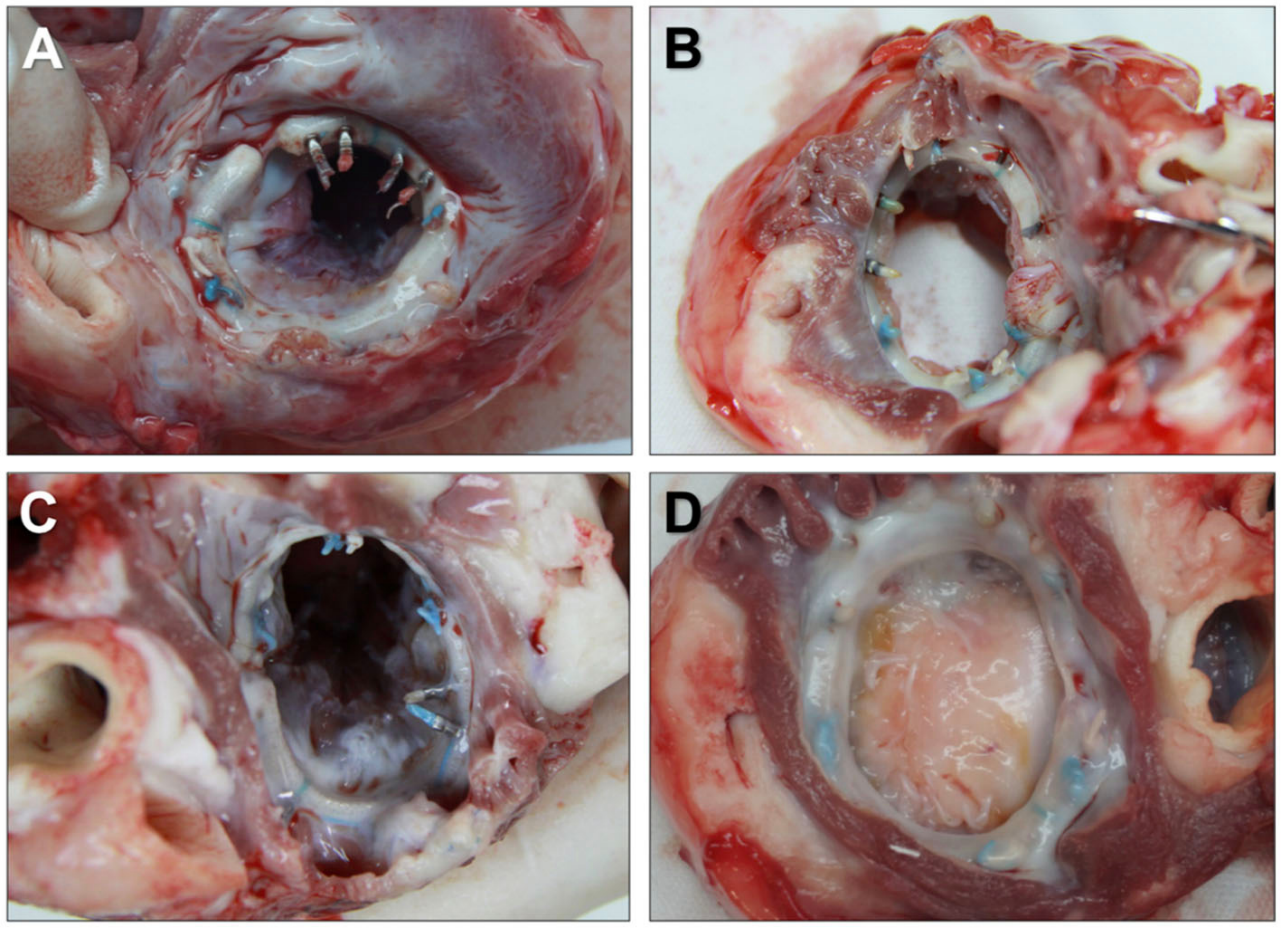
Representative *in situ* view of the left atrium at the implantation site of the annuloplasty ring. Annuloplasty rings were tightly secured by 6 Easy-Knot clips and 6 hand-made knots without any disengagement or displacement at indicated time points. Day 30 **(A)**, Day 60 **(B)**, Day 90 **(C)**, and Day 180 **(D)**.

The firmness of each knot was evaluated by gross examination. The representative *in situ* view of the left atrium at each endpoint is shown in Figure 4. No peri-leakage was found around the Easy-Knot clips, whereas one was observed aside from a hand-made knot in the Day-90 group (Supplementary Figure S1). All mitral valves were found intact without defect, stenosis, prolapse, insufficiency, or perforation around the knotting sites.

**Figure 5:**
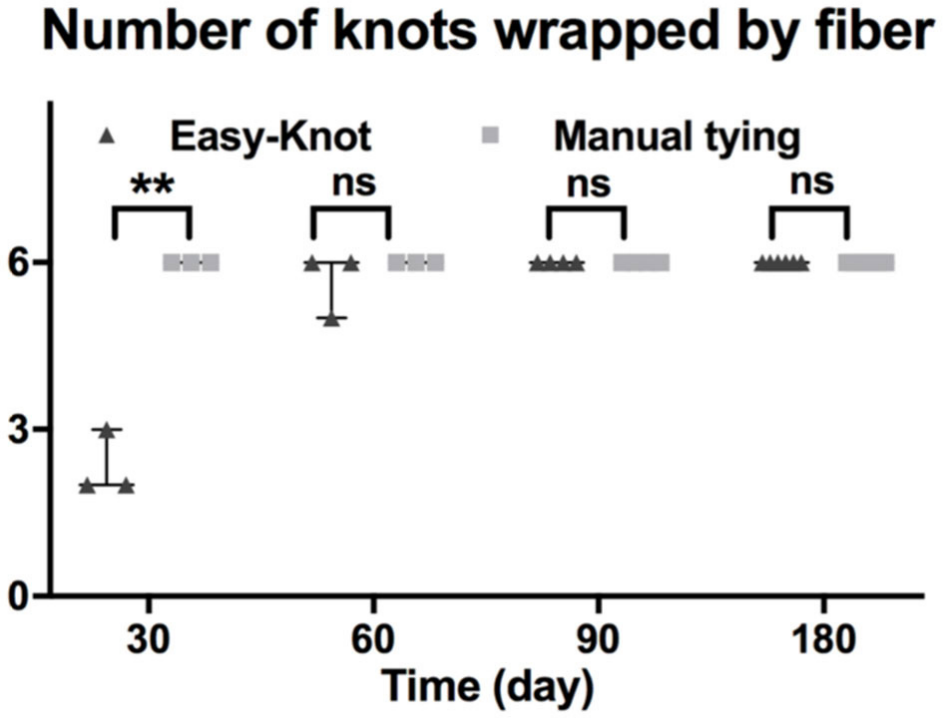
The number of knots wrapped by fiber at indicated time points. **p < 0.01.

The number of knots wrapped by fiber was counted during the gross examination. As shown in Figure 5, all the manual-tying knots were wrapped by fiber by Day 30. On the contrary, only 7 of 18 Easy-Knot clips were wrapped by fiber by Day 30. However, all the Easy-Knot clips were gradually wrapped by fiber by Day 90.

**Figure 6:**
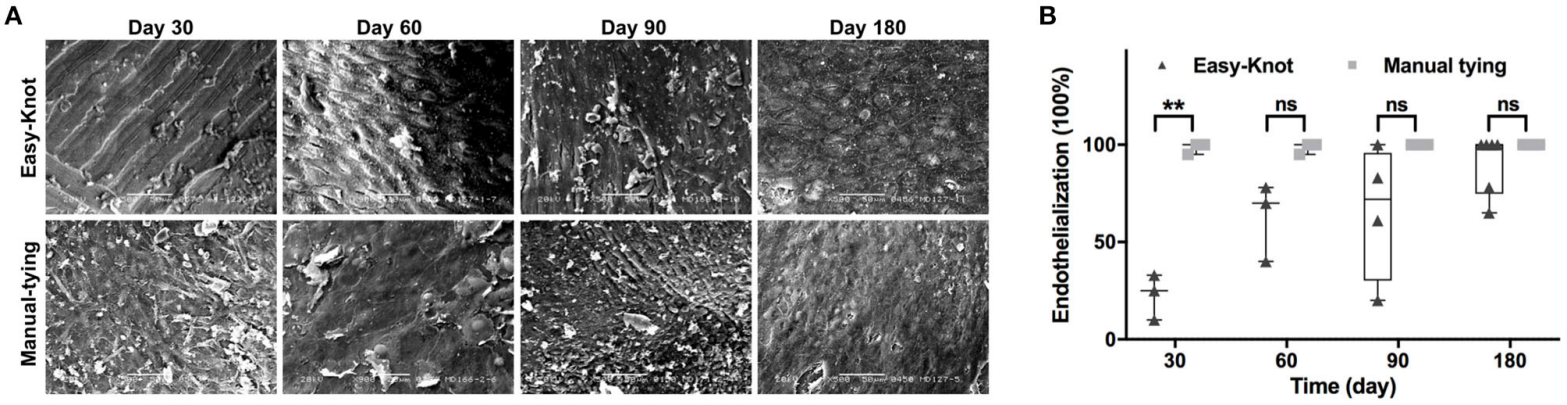
The ultrastructure of knotting site surface under scanning electron microscope (SEM) and endothelialization. *p < 0.05; **p < 0.01; ns, not significant. **(A)** The dynamic change of the ultrastructure of endothelial cell coating on the metal surface of titanium clip or the manual-tying knotting site under SEM at indicated time points. **(B)** Statistical analysis of the percentage of endothelialization in the Easy-Knot group and the manual-tying group. *p < 0.05; **, *p* < 0.01; ns, not significant.

Endothelialization was evaluated by SEM and calculated according to the formula: endothelialization % = (neointimal area/material area) × 100%. As shown in Figure 6A, the metal texture was obvious on the surface of the Easy-Knot clip at Day 30 but got endothelialized overtime. At Day 180, most clips were completely covered by endothelial cells with no significant difference compared with the control group (Figure 6B).

**Figure 7:**
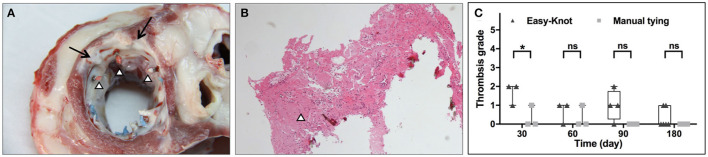
Representative gross and histological view of thrombi at knotting site at Day 180 and thrombosis grade at indicated time points. **(A)** Morphology of the left atrial surface at the implantation site of the annuloplasty ring. Two of the six titanium clips were completely coated with proliferative fibrous tissue (↑), red thrombosis was observed at 3 thread ends (Δ). **(B)** Hematoxylin-eosin (H&E) staining of the thread end of a titanium clip (Δ). **(C)** Thrombosis grade of the Easy-knot group and the manual-tying group at indicated time points. *p < 0.05; **p < 0.01; ns, not significant.

Thrombosis was evaluated by gross and histological examinations. As shown in Figures 7A,B, small red thrombi were found on the thread end of the suture for both groups. No thrombus was found on the surface of the Easy-Knot clip. The thrombosis rate of each group was calculated according to the formula: thrombosis rate = (number of knots with thrombus/number of total knots) × 100%. At Day 30, 77.8% (14/18) cases in the Easy-Knot group and 5.56% (1/18) cases in the control group were with thrombus. For longer follow-up time, 27.78% (5/18) vs. 5.56% (1/18) cases and 37.50% (9/24) vs. 0.00% (0/24) cases in Easy-Knot vs. control groups were found with thrombus at Day 60 and Day 90, respectively. At Day 180, 13.89% (5/36) cases in the Easy-Knot showed thrombus and no thrombus was observed in the control group. The thrombi were graded based on the size according to the biological test criteria ‘GB/T 14233.2-2005' Appendix B (Supplementary Table S2). As shown in Figure 7C, the Easy-Knot group manifested bigger thrombi than the control group at Day 30. At Day 180, the Easy-Knot group became comparable with the control (*p* > 0.05). All the thrombi were found small with a mean diameter < 5 mm and were graded lower than 2, which means that the Easy-Knot was within the acceptable range for antithrombotic property according to the ‘GB/T 14233.2-2005' Appendix B.

**Figure 8:**
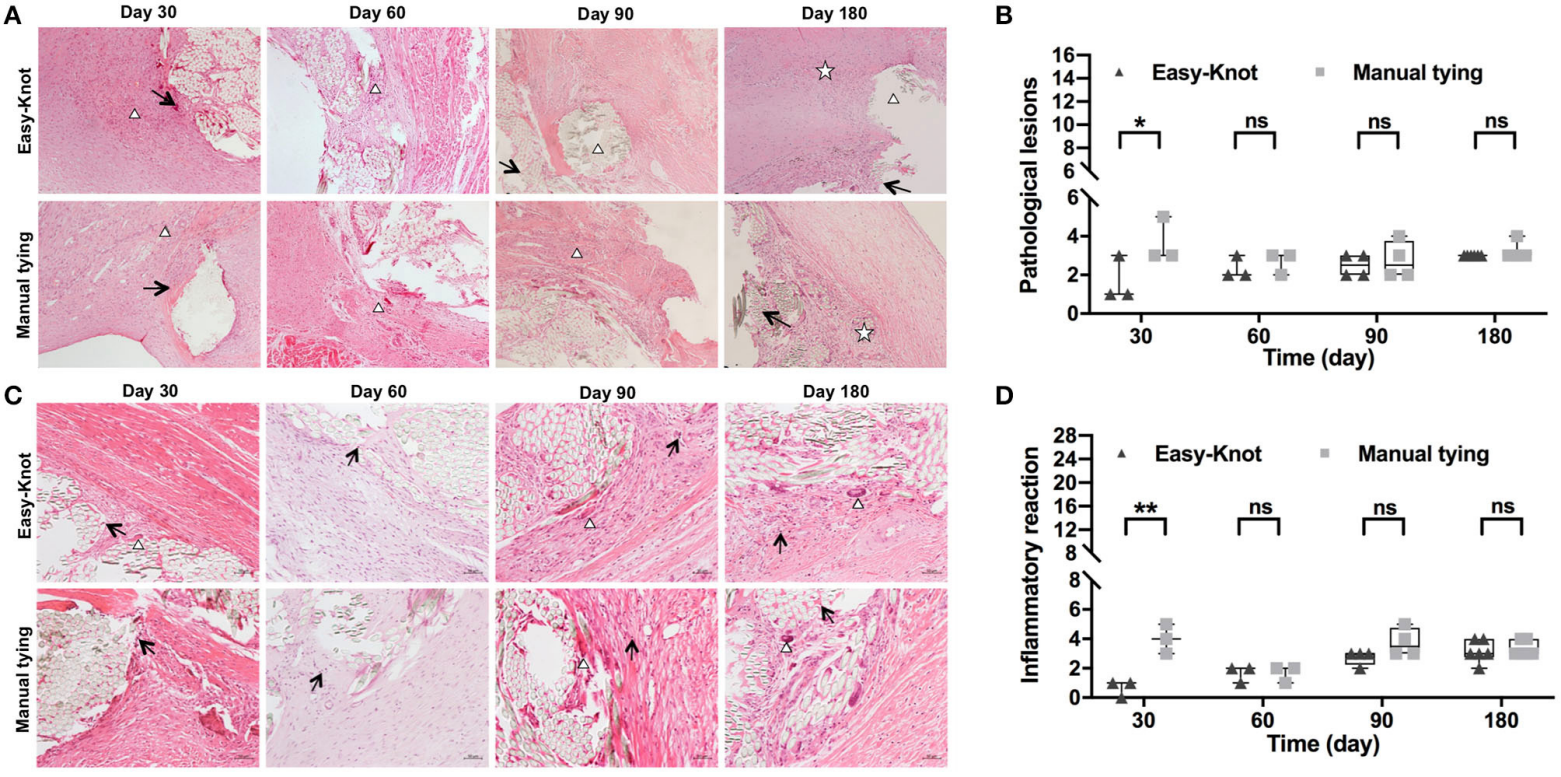
Histopathological examination of tissue around the annuloplasty ring at the indicated time. **(A)** Histopathological examination showed the typical microstructure of the sutures at indicated time points. Δ, fibrous tissue with granulomatous reaction; ↑, slight calcification; ✰, chronic inflammation response. **(B)** Statistical analyses of pathological lesions. **(C)** Histopathological examination showed the typical inflammatory cells↑, lymphocyte; Δ, monocyte macrophage. **(D)** Statistical analyses of pathological lesions. *p < 0.05; **p < 0.01, ns; not significant.

Local tissue pathological lesions and inflammation were evaluated through histological examination. The sum of the score of 4 items, such as (i) neovascularization, (ii) fibrosis, (iii) fat infiltration, and (iv) calcification (0–4 points for each item) was calculated to evaluate the pathological lesions. As shown in Figures 8A,B, slight fibroplasias, granuloma, and calcification were observed in both groups at Day 30. In addition, necrosis was observed in the control group, indicating a slightly more severe lesion. On Day 60, a comparable level of granuloma was observed in both groups. On Day 90, chronic inflammation was observed around the annuloplasty ring and at the knot sites of the Easy-Knot group, while granulomatous inflammation of foreign bodies and inflammatory cell infiltration were obviously observed in the control group. On Day 180, chronic inflammation was observed in both groups. The sum of the score of 7 items, such as (i) neutrophils, (ii) lymphocytes, (iii) eosinophils, (iv) plasma cells, (v) giant cells, (vi) macrophages, and (vii) tissue necrosis (0–4 points for each item) was calculated to evaluate the inflammation reaction. As shown in Figures 8C,D, the Easy-Knot group manifested a significantly lower inflammatory reaction at Day 30 and became comparable with the control group for the longer follow-up time. The CD45 staining showed the same pattern (Supplementary Figure S2).

**Figure 9:**
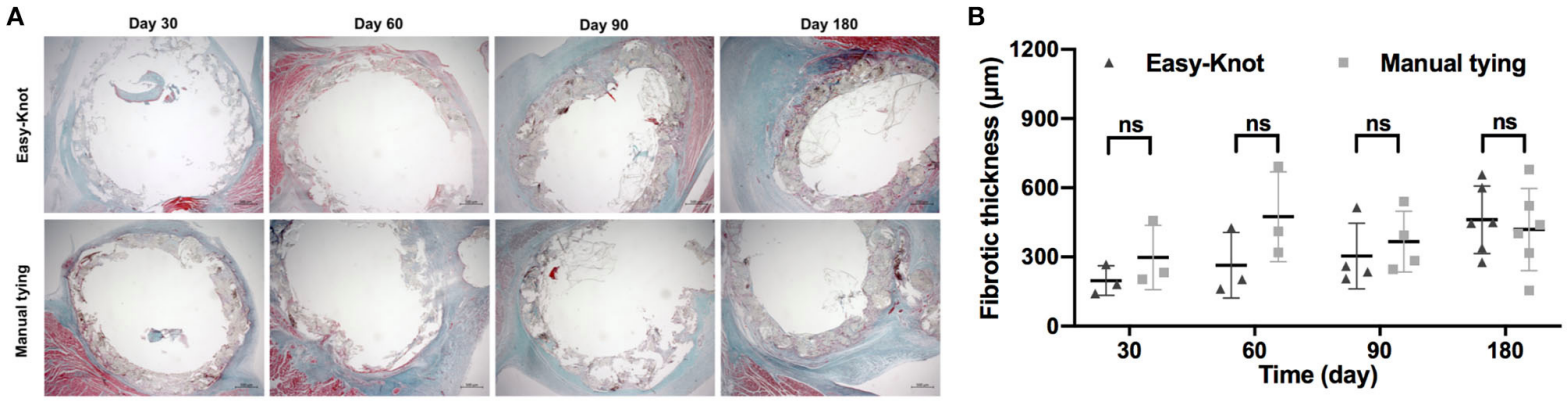
**(A)** Masson trichrome staining of tissue around the annuloplasty ring at indicated time points after implantation and **(B)** fibrotic thickness analysis. ns; not significant.

Fibrotic thickness was analyzed based on Masson trichrome staining. As shown in Figure 9, comparable fibrosis was detected in both groups.

**Figure 10:**
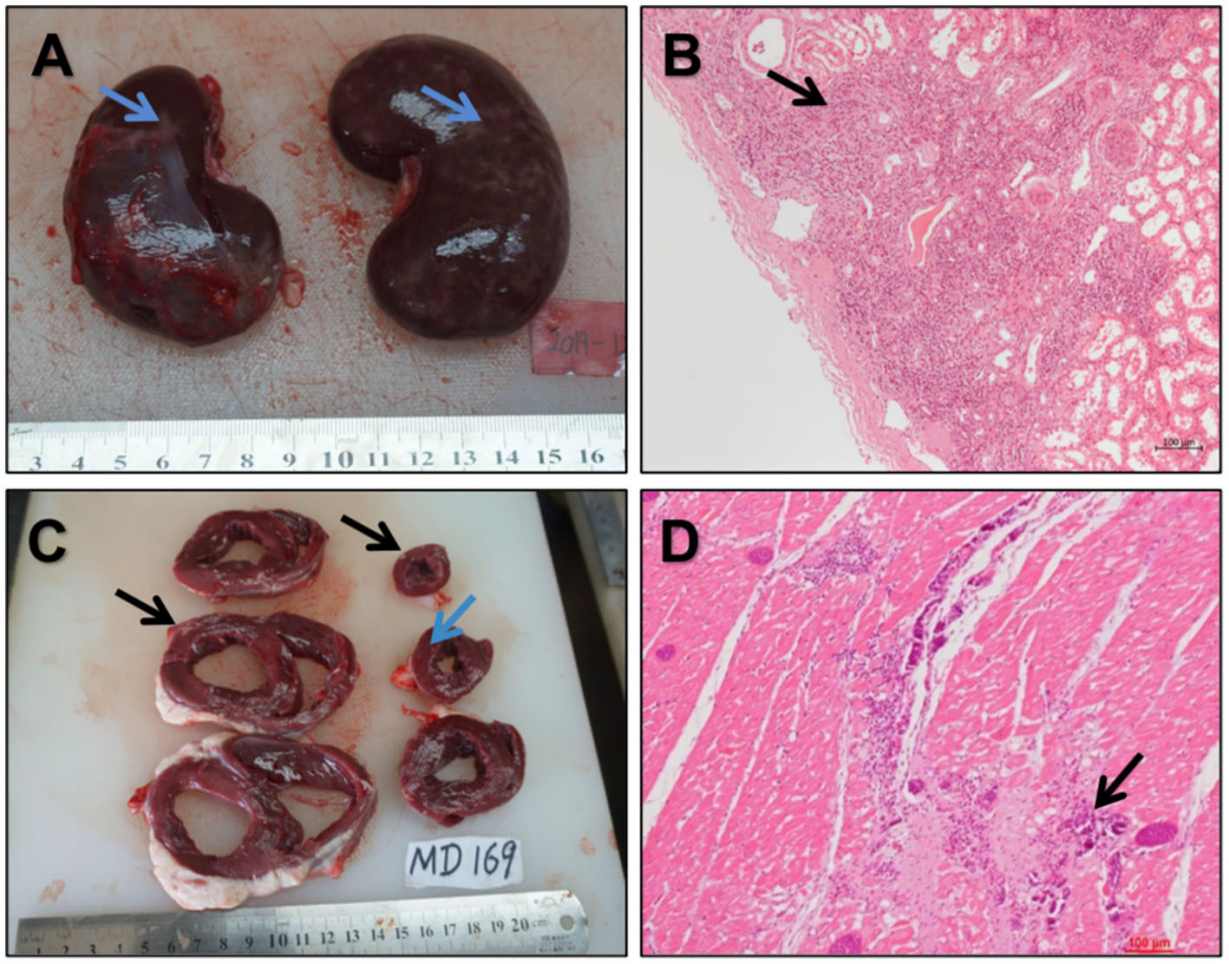
Gross and histological views of the vital organs **(A,B)**. Gross **(A)** and histological **(B)** views of diffused renal infarction (↑) **(C,D)**. Anatomic structures of multifocal fibrous scar **(C)** (↑) and histopathological examination **(D)** of multifocal fibrous scar and calcification in the myocardium (↑).

For the 16 enrolled sheep, four visceral complications occurred (Figure 10): 1 case of diffuse renal infarction (area ratio = 20%) and 1 case of small focal renal infarction (area ratio < 5%), both caused by thromboembolism, 1 case of small cardiac infarction caused by intraoperative air embolism (area ratio < 5%), and 1 case of myocardial ischemia reperfusion injury.

Collectively, our data confirmed the safety of the Easy-Knot in the preclinical studies.

## Discussion

A variety of tying knot devices have been developed by different medical corporations, such as Autosuture Endo Stitch (Covidien, Mansfield, MA, USA) (20), Suture Assist (Ethicon Endo-Surgery, Cincinnati, OH, USA) (21), and Suture Cartridge (22) to reduce the procedure time. These devices employed various strategies, such as the needle holder knotting method (23), flat needle (21), and suture optimization (24). However, the majority of fastening devices and knotting methods are confronted with limitations, such as cumbersome, requirement of special storage, difficulty to operate, and low practicability preventing them from being popularly applied for cardiac surgeries. The automated titanium suture fastener stood out and became widely accepted because of its time saving, simplicity, and reliability (25–27). Furthermore, the 30 cm-long outer tube facilitates its use for remote knot tying in minimally invasive valve surgery (7, 16, 28, 29).

In this study, we employed a self-control method, that the annuloplasty ring was secured by both the automated fastener and manual tying in the same ovine individual, to evaluate the safety and effectiveness outcomes of the Easy-Knot. In this way, the evaluated aspects, such as knotting time, firmness of the knots, endothelialization, thrombosis, and local tissue reactions could be clearly discriminated between the two groups, meanwhile, the interference of individual differences was eliminated to the most extent.

Thrombosis is the critical criterion for safety evaluation. For surgeries that introduce metal implants, a thrombus would form on the surface of the metal in some clinical complications (30). Meanwhile, it has been reported that the fallen pieces of the metal implants migrated to distal organs and form metallic emboli (31). In our study, the Easy-knot performed outstandingly at these aspects: no thrombus formed on the surface of the clip, and the distal emboli were not formed from metallic pieces. However, thrombi were found on the thread ends of the suture. To our knowledge, allogenetic transfusion and CBP could be highly speculative for the embolism in the ovine. On the other hand, the decompaction of the braided suture fibers after trimming could be a pivot point for embolism. Compared with the cutting off of the suture by scissors in the control group, suture in the Easy-Knot group was sheared off by the blade in the front of the device, which may cause the decompaction. To solve the thromboembolism problem, the improvement on the trimming method and the suture material is ongoing.

The other focus of this study was to evaluate the effectiveness of the Easy-Knot. Our result showed that the Easy-Knot was an effective and easy-to-use device because of its time saving, simplicity, reliability, and consistency. The average time cost for the entire knotting procedure was 22.67 vs. 57.79 s for the Easy-Knot vs. manual tying. Since the sewing procedure was the same for both groups, the time cost without this procedure was recorded separately. Deploying of the Easy-Knot clip and squeezing of the trigger cost only 3.6 s, which was one-eighth of the time cost for traditional manual tying. Meanwhile, gross anatomy showed that the one side of the annuloplasty rings fixed by the Easy-Knot was firm without detachment, displacement, or leakage with no significant differences compared with the control side. Therefore, the Easy-Knot significantly shortened the knotting time without compromising the firmness of the knots and operational accuracy.

As more and more new cardiovascular devices and techniques are emerging, there is an urgent need for standardized and specialized pre-clinical *in vivo* evaluation methods for the implanted cardiovascular devices. In this study, by incorporating the criteria of the Chinese national drug supervision and administration of medical devices technical evaluation center (NMPA) and the U.S. Food and Drug Administration (FDA), together with the long experience of our institution at the *in vivo* preclinical evaluation for cardiovascular implant materials, we provided a reference evaluation system for the automated titanium suture fastener or similar devices in cardiac valvular surgery.

In conclusion, the Easy-Knot significantly shortened the time for tying knots for the fixation of the annuloplasty ring in the ovine model, with comparable safety and effectiveness as manual tying.

## Data Availability Statement

The original contributions presented in the study are included in the article/Supplementary Material, further inquiries can be directed to the corresponding author/s.

## Ethics Statement

The animal study was reviewed and approved by Ethical Committee of Fuwai Hospital, Chinese Academy of Medical Sciences and Peking Union Medical College.

## Author Contributions

BL and GY contributed to the collection and assembly of data. JZ, MZ, BY, JL, and YS contributed to the collection and assembly of data and manuscript writing. XW, BL, SB, and LZ contributed to the conception and design, data analysis and interpretation, manuscript writing, and revision. All the authors finally approved the manuscript.

## Conflict of Interest

The authors declare that the research was conducted in the absence of any commercial or financial relationships that could be construed as a potential conflict of interest.

## Publisher's Note

All claims expressed in this article are solely those of the authors and do not necessarily represent those of their affiliated organizations, or those of the publisher, the editors and the reviewers. Any product that may be evaluated in this article, or claim that may be made by its manufacturer, is not guaranteed or endorsed by the publisher.

## Abbreviations

CPB: cardiopulmonary bypass
ACC: aortic cross-clamp
SEM: scanning electron microscope
ACT: activated clotting time
RBC: red blood cell count
WBC: white blood cell count
HGB: hemoglobin
PLT: platelet
LDH: lactic dehydrogenase
CK: creatine kinase
CRE: creatinine
BUN: blood urea nitrogen
ALT: alanine aminotransferase
AST: aspartate transaminase
PT: prothrombin time
INR: international normalized ratio
and APTT: activated partial thromboplastin time.
