# Supplementary material for: Safety and Effectiveness Outcomes of a Novel Automated Titanium Suture Fastener Device Applied for Heart Valve Surgery in an Ovine Model
Source: Front Cardiovasc Med. 2022 Feb 11;9:783208. doi: 10.3389/fcvm.2022.783208 (PMC8874148; doi:10.3389/fcvm.2022.783208)
Supplement: Supplementary file 1 [file Data_Sheet_1.PDF]

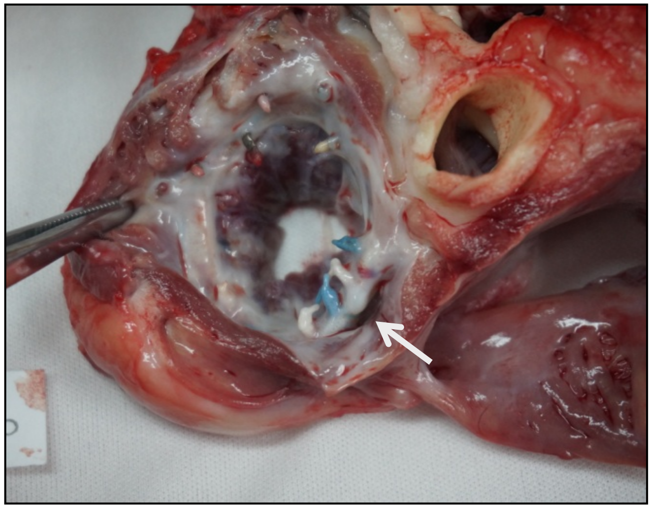

Figure S1. A peri-leakage aside a manual-tying knot at Day 90 (↑).

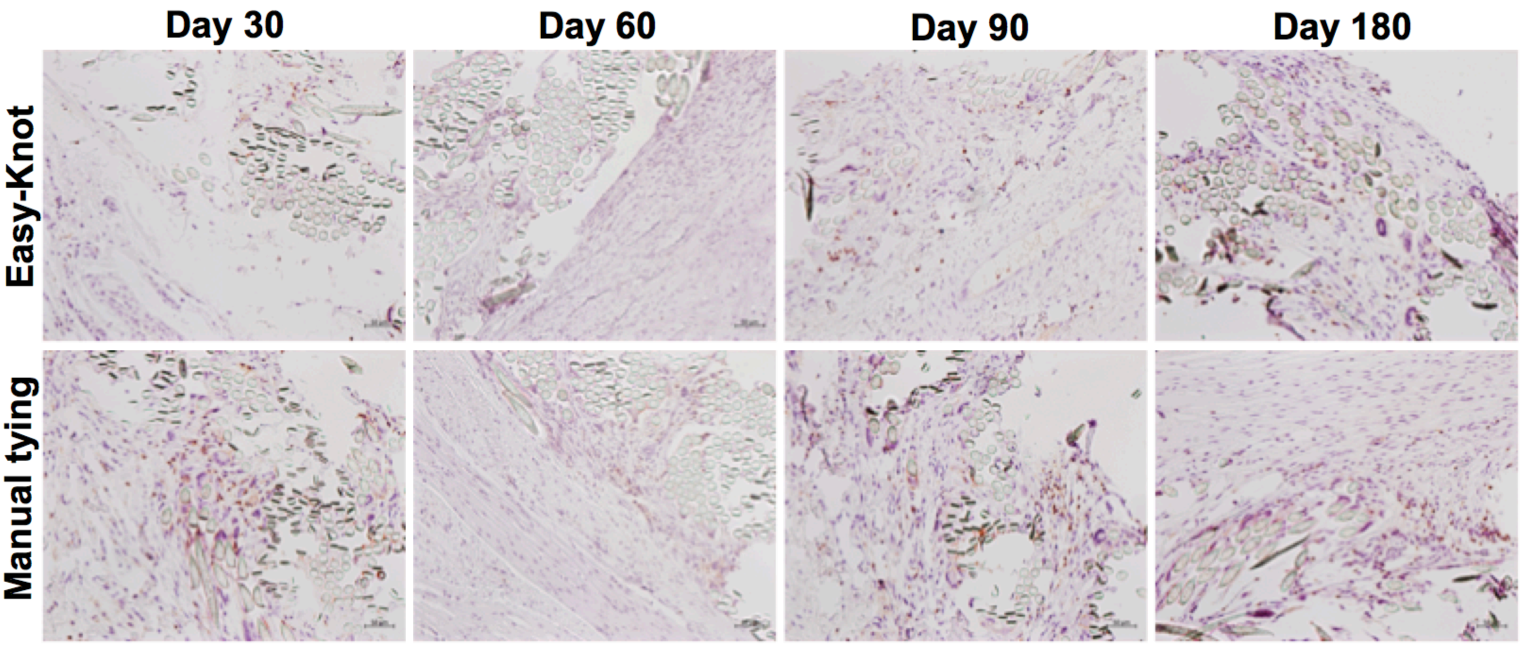

Figure S2. Representative IHC images of tissue around the annuloplasty ring at indicated time points for inflammatory reaction analysis. *, P<0.05.
