# Supplementary material for: Safety and Effectiveness Outcomes of a Novel Automated Titanium Suture Fastener Device Applied for Heart Valve Surgery in an Ovine Model
Source: Front Cardiovasc Med. 2022 Feb 11;9:783208. doi: 10.3389/fcvm.2022.783208 (PMC8874148; doi:10.3389/fcvm.2022.783208)
Supplement: Supplementary file 2 [file Data_Sheet_2.docx]

**Tables**

**Table S1.** Basic information of animals

|  | **No.** | **Gender** | **Weight (kg)** | **Group** | **Survival time (day)** |
| --- | --- | --- | --- | --- | --- |
| 1 | S2019-123 | F | 47 | Day 30 | 30 |
| 2 | S2019-124 | F | 50 | Day 30 | 30 |
| 3 | S2019-169 | F | 52 | Day 30 | 30 |
| 4 | S2019-165 | F | 46 | Day 60 | 64 |
| 5 | S2019-166 | F | 59 | Day 60 | 63 |
| 6 | S2019-167 | F | 47 | Day 60 | 64 |
| 7 | S2019-164 | F | 55 | Day 90 | 92 |
| 8 | S2019-168 | F | 53 | Day 90 | 90 |
| 9 | S2019-170 | F | 52 | Day 90 | 90 |
| 10 | S2019-171 | F | 46 | Day 90 | 90 |
| 11 | S2019-125 | F | 46 | Day 180 | 182 |
| 12 | S2019-126 | F | 46 | Day 180 | 182 |
| 13 | S2019-127 | M | 39 | Day 180 | 181 |
| 14 | S2019-128 | M | 40 | Day 180 | 182 |
| 15 | S2019-129 | F | 43 | Day 180 | 182 |
| 16 | S2019-130 | F | 44 | Day 180 | 182 |

Note：M = Male， F = Femal.

**Table S2.** Classification criteria for thrombosis response (GB/T14233.2-2005)

| **Thrombus level** | **Thrombosis observation** |
| --- | --- |
| 0 | Free of Thrombosis |
| 1 | Minor thrombosis, a blood clot or a very thin blood clot in one place |
| 2 | Minor thrombosis, tiny blood clots in several places |
| 3 | Moderate thrombosis, the blood clot covered less than half the length of the implanted sample |
| 4 | Severe thrombosis, the blood clot covered more than half the length of the implanted sample |
| 5 | Vascular occlusion |
